# Cross-Cultural Differences in the Processing of Non-Verbal Affective Vocalizations by Japanese and Canadian Listeners

**DOI:** 10.3389/fpsyg.2013.00105

**Published:** 2013-03-19

**Authors:** Michihiko Koeda, Pascal Belin, Tomoko Hama, Tadashi Masuda, Masato Matsuura, Yoshiro Okubo

**Affiliations:** ^1^Department of Neuropsychiatry, Nippon Medical SchoolTokyo, Japan; ^2^Voice Neurocognition Laboratory, Institute of Neuroscience and Psychology, College of Medical, Veterinary and Life Sciences, University of GlasgowGlasgow, UK; ^3^Department of Biofunctional Informatics, Tokyo Medical and Dental UniversityTokyo, Japan; ^4^Division of Human Support System, Faculty of Symbiotic Systems Science, Fukushima UniversityFukushima, Japan

**Keywords:** montreal affective voices, emotion, voice, cross-cultural differences, social cognition

## Abstract

The Montreal Affective Voices (MAVs) consist of a database of non-verbal affect bursts portrayed by Canadian actors, and high recognitions accuracies were observed in Canadian listeners. Whether listeners from other cultures would be as accurate is unclear. We tested for cross-cultural differences in perception of the MAVs: Japanese listeners were asked to rate the MAVs on several affective dimensions and ratings were compared to those obtained by Canadian listeners. Significant Group × Emotion interactions were observed for ratings of Intensity, Valence, and Arousal. Whereas Intensity and Valence ratings did not differ across cultural groups for sad and happy vocalizations, they were significantly less intense and less negative in Japanese listeners for angry, disgusted, and fearful vocalizations. Similarly, pleased vocalizations were rated as less intense and less positive by Japanese listeners. These results demonstrate important cross-cultural differences in affective perception not just of non-verbal vocalizations expressing positive affect (Sauter et al., 2010), but also of vocalizations expressing basic negative emotions.

## Introduction

Vocal affective processing has an important role in ensuring smooth communication during human social interaction as well as facial affective processing. Facial expressions are generally recognized as the universal language of emotion (Ekman and Friesen, 1971; Ekman et al., 1987; Ekman, 1994; Izard, 1994; Jack et al., 2012): however, several studies have demonstrated cross-cultural differences in facial expression between Western and Eastern groups (Ekman and Friesen, 1971; Ekman et al., 1987; Matsumoto and Ekman, 1989; Izard, 1994; Yrizarry et al., 1998; Elfenbein and Ambady, 2002; Jack et al., 2009, 2012). Whether such cross-cultural differences also exist in the recognition of emotional vocalizations is not clear.

Most previous cross-cultural studies of auditory perception have investigated the processing of emotional Valence using word stimuli (Scherer and Wallbott, 1994; Kitayama and Ishii, 2002; Ishii et al., 2003; Min and Schirmer, 2011). One important study demonstrated cross-cultural differences in the rating of Intensity when subjects recognized meaning of the words with major emotions such as joy, fear, anger, sadness, and disgust (Scherer and Wallbott, 1994). Another previous study examined cross-cultural differences in the perception of emotional words (Kitayama and Ishii, 2002). This study indicated that native English speakers spontaneously pay more attention to verbal content than to vocal tone when they recognize emotional words, whereas native Japanese speakers spontaneously attend more to vocal tone than to verbal content. The other study has shown that Japanese are more sensitive to vocal tone compared to Dutch participants in the experiment of the multisensory perception of emotion (Tanaka et al., 2010). Further, one other study demonstrated cross-cultural differences in semantic processing of emotional words (Min and Schirmer, 2011), but found no difference in the processing of emotional prosody between native and non-native listeners. These studies suggest cross-cultural differences in auditory recognition of emotional words.

Studies of affective perception in speech prosody are made complex, in particular, by the potential interactions between the affective and the linguistic contents of speech (Scherer et al., 1984; Murray and Arnott, 1993; Banse and Scherer, 1996; Juslin and Laukka, 2003). To avoid this interaction, some studies have controlled the processing of semantic content using pseudo-words (Murray and Arnott, 1993; Schirmer et al., 2005) or pseudo-sentences (Ekman and Friesen, 1971; Pannekamp et al., 2005; Schirmer et al., 2005). The other previous study has employed a set of low-pass filtered vocal stimuli to select the final set of emotional utterances (Ishii et al., 2003), i.e., non-verbal vocalizations often accompanying strong emotional states such as laughs or screams of fear. Non-verbal affective vocalizations are ideally suited to investigations of cross-cultural differences in the perception of affective information in the voice since they eliminate the need to account for language differences between groups.

A recent study compared the perception of such non-verbal affective vocalizations by listeners from two highly different cultures: Westerners vs. inhabitants of remote Namibian villages. Non-verbal vocalizations expressing negative emotions could be recognized by the other culture much better than those expressing positive emotions, which lead the authors to propose that a number of primarily negative emotions have vocalizations that can be recognized across cultures while most positive emotions are communicated with culture-specific signals (Sauter et al., 2010). However this difference could be specific to English vs. Namibian groups, reflecting for instance different amounts of exposure to vocalizations through media or social interactions, and might not generalize to other cultures.

In the present experiment we tested for cross-cultural differences in perception of affective vocalizations between two cultures much more comparable in socio-economic status and exposure to vocalizations: Canadian vs. Japanese participants. Stimuli consisted of the Montreal Affective Voices (MAVs; Belin et al., 2008), a set of 90 non-verbal affect bursts produced by 10 actors and corresponding to emotions of Anger, Disgust, Fear, Pain, Sadness, Surprise, Happiness, and Pleasure. The MAVs have been validated in a sample of Canadian listeners and showed high inter-reliability in judgments of emotional Intensity, Valence, and Arousal as well as hit rates in emotional recognition (Belin et al., 2008). Here, we collected affective ratings using similar procedures in Japanese listeners and compared those ratings to those obtained in the Canadian listeners. Before the experiment, we predicted that ratings of negative emotion are culturally universal although cross-cultural differences would exist in ratings of positive emotion.

## Materials and Methods

### Subjects

Thirty Japanese subjects (male 15, female 15) participated in this study. The average age was 22.3 ± 1.4 years. The educational years of Japanese subjects were 14.1 ± 0.3. The data of Japanese subjects were compared with 29 Canadian subjects (male 14, female 15); average age: 23.3 ± 1.5 years (Belin et al., 2008). Both Japanese and Canadian participants consisted exclusively of undergraduate students.

After a thorough explanation of the study, written informed consent was obtained from all subjects, and the study was approved by the Ethics Committee of Nippon Medical School.

### Voice materials

The MAVs: 10 French-Canadian actors expressed specific emotional vocalizations and non-emotional vocalizations (neutral sounds) using “ah” sounds. The eight emotional vocalizations were angry, disgusted, fearful, painful, sad, surprised, happy, and pleased. The simple “ah” sounds were used to control the influence of lexical-semantic processing. Since each of the eight emotional vocalizations and the neutral vocalization were spoken by 10 actors, the total number of MAVs sounds was 90. The MAVs are available at: http://vnl.psy.gla.ac.uk/

### Evaluation scale

Each emotional vocalization was evaluated using three criteria: perceived emotional Intensity in each of the eight Emotions, perceived Valence, and perceived Arousal. Each scale had a range from 0 to 100.

The Valence scale represented the extent of positive or negative emotion expressed by the vocalization: 0 was extremely negative, and 100 was extremely positive. The Arousal scale represented the extent of excitement expressed by the vocalization: 0 was extremely calm, and 100 was extremely excited. The Intensity scale represented the Intensity of a given emotion expressed by the vocalization: 0 was not at all intense, and 100 was extremely intense. The Intensity scale was used for eight emotions: Anger, Disgust, Fear, Pain, Sadness, Surprise, Happiness, and Pleasure.

### Methods of evaluation by participants

The MAVs vocalizations were played on a computer in a pseudo-random order. The subjects listened with headphones at a comfortable hearing level, and they evaluated each emotional vocalization for perceived Intensity, Valence, and Arousal using a visual analog scale in English on a computer (10 ratings per vocalization: 8 Intensity ratings, 1 Valence rating, 1 Arousal rating). Simultaneously, participants were given a printed Japanese translation of the scale labels, and by referring to this Japanese sheet, the test was performed using exactly the same procedure as in the Canadian study (Belin et al., 2008). All Japanese participants performed the experiment using a translation sheet with emotional words translated from English to Japanese. Based on previous studies (Scherer and Wallbott, 1994), the Japanese translation of English emotional labels was independently assessed by three clinical psychologists. Through their discussion, the appropriate emotional labels were determined.

### Statistical analysis

Statistical calculations were made using SPSS (Statistical Package for Social Science) Version 19.0. The Japanese data and the Canadian published data, with permission to verify, were statistically analyzed. A previous study demonstrated gender effects in Canadian participants using the MAV (Belin et al., 2008). Using the same methods to reveal the gender effects, an ANOVA with Emotion, Actor gender, and Participant gender as factors was calculated for ratings by the Japanese listeners. Further, to clarify the cross-cultural effect between Japanese and Canadian participants, three mixed two-way ANOVAs were calculated on ratings of Intensity, Valence, and Arousal. For each mixed ANOVA, to verify the equality of the variance of the differences by Emotions, Mauchly’s sphericity was calculated. If the sphericity could not be assumed using Mauchly’s test, Greenhouse–Geisser’s correction was calculated.

### Reliability and accuracy

First, we analyzed the inter-subject reliability of the ratings using Cronbach’s alpha. Next, we examined the Intensity ratings for their sensitivity (hit rate, by Emotion) and specificity (correct rejection rate, by rating scale). Based on the previous report (Belin et al., 2008), the accuracy of emotional recognition was investigated using measures of sensitivity (hit rate, by Emotion) and specificity (correct rejection rate, by rating scale). For each vocalization, participants rated the perceived emotional Intensity along each of eight different scales (Anger, Disgust, Fear, Pain, Sadness, Surprise, Happiness, and Pleasure). To calculate sensitivity, for a given portrayed emotion, a maximum Intensity rating in the corresponding scale (i.e., if Intensity rating of Anger was highest when the subject listened to angry vocalization) was taken as a hit; otherwise, as a miss. In other words, emotions with high hit rates are those that are well recognized, i.e., that scored highest on the scale of the intended emotion. Conversely, specificity relates to the extent to which the rating scale measures what it is intended to measure. To calculate specificity for a given rating scale, if the maximum score was obtained for the corresponding portrayed emotion across the eight vocalizations from one actor (i.e., when the subject listened to disgusted vocalization by actor 1, if rating of Disgust was highest in the eight emotional items), it was taken as a correct rejection; otherwise, as a false alarm. A highly specific rating scale is one rating scale for which the corresponding vocalization obtains the highest score. In other words, it is a measure of how a rating scale is specific to an emotion.

## Results

### Affective rating

**Figure 1:**
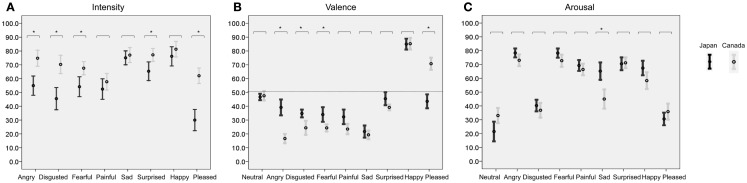
**Shows the distribution of ratings (error bar: mean ± SD) for each emotional sound judged by 30 Japanese and 30 Canadian participants for (A) Intensity, (B) Valence, and (C) Arousal**. Each horizontal axis represents each rating score (0–100). Each vertical axis shows categories of emotional vocalizations. A mixed two-way ANOVA represents significant main effects of Subject group (Japanese and Canadian) and Emotion, respectively: *p* < 0.001; *p < 0.05/8 (Intensity), *p < 0.05/9 (Valence), *p < 0.05/9 (Arousal); *post hoc* *t*-test.

Inter-participant (30 participants) reliability across the 90 items [10 ratings scales: (Valence, Arousal, eight emotional Intensities) × (9 Emotional sounds) was analyzed: Cronbach’s alpha = Japanese: 0.941, *F*(89, 299) = 230.6, *p* < 0.001]. Since this reliability for 30 subjects is very high, the ratings of 10 actors’ vocalizations were averaged with the ratings of all 30 Japanese participants. [Canadian participants had an inter-participant reliability rating of 0.978 (Belin et al., 2008)]. Table 1 shows the averaged ratings of Intensity, Valence, and Arousal for the present sample of Japanese participants and the Canadian participants in the study of Belin et al. (2008). Figure 1 shows the distribution (average ± 2 SD) of ratings of 1-1. Intensity, 1-2. Valence, and 1-3. Arousal in Japanese and Canadian participants.

**Table 1:** **The mean (*M*) ratings of 1. Intensity, 2. Valence, and 3. Arousal for 10 actors’ voices (5 male actors, 5 female actors) by 30 Japanese and 30 Canadian participants**.

1-1: Intensity						1-2: Valence						1-3: Arousal					
Vocal expression	Japan		Canada		*t*-test	Vocal expression	Japan		Canada		*t*-test	Vocal expression	Japan		Canada		*t*-test
	*M*	SEM	*M*	SEM	*P*		*M*	SEM	*M*	SEM	*P*		*M*	*SEM*	*M*	*SEM*	*P*
Neutral	–	–	–	–		Neutral	46.7	1.2	47.6	1.7	n.s.	Neutral	21.5	3.6	33.0	2.7	n.s.
Angry	55.0	3.5	74.7	2.9	<0.001*	Angry	39.2	2.9	16.6	1.7	<0.001*	Angry	78.3	1.6	73.0	2.1	n.s.
Disgusted	45.5	4.0	70.3	3.3	<0.001*	Disgusted	34.8	1.4	24.4	2.5	<0.001*	Disgusted	40.3	2.1	36.9	2.7	n.s.
Fearful	54.1	3.6	67.6	2.4	0.003*	Fearful	34.0	2.7	24.3	1.3	0.002*	Fearful	78.3	1.7	72.6	2.2	n.s.
Painful	52.4	3.7	57.8	3.0	n.s.	Painful	32.3	2.7	23.5	1.9	n.s.	Painful	69.3	2.0	66.4	2.1	n.s.
Sad	75.1	2.5	77.0	2.7	n.s.	Sad	21.7	2.2	19.3	1.6	n.s.	Sad	65.2	3.1	45.0	3.5	<0.001*
Surprised	65.4	3.4	77.2	2.4	0.006*	Surprised	45.4	2.3	39.1	1.1	n.s.	Surprised	70.5	2.3	71.2	1.9	n.s.
Happy	76.2	3.5	81.3	2.8	n.s.	Happy	84.9	2.0	85.2	2.1	n.s.	Happy	67.4	2.6	58.3	3.1	n.s.
Pleased	30.0	3.8	62.1	2.8	<0.001*	Pleased	43.5	2.6	70.8	2.2	<0.001*	Pleased	30.6	2.3	35.9	2.9	n.s.

SEM is mean of standard deviation. A mixed two-way ANOVA demonstrated significant main effects of Group and Emotion in Intensity and Valence: *p* < 0.001; *represents significant difference of *post hoc* tests; Intensity: *p* < 0.05/8, Valence: *p* < 0.05/9, Arousal: *p* < 0.05/9.

### Intensity

A mixed two-way ANOVA with listeners’ Group (Japanese, Canadian) and Emotion (*n* = 8) as factors was calculated on Intensity scores. A significant main effect was revealed between listener’s Groups [*F*(1, 57) = 20.828, *p* < 0.001] as well as among the Emotions [*F*(5.5, 313.5) = 40.520, *p* < 0.001; Greenhouse–Geisser’s test]. Crucially, a significant interaction between Group and Emotion was observed, *F*(5.5, 313.5) = 9.137, *p* < 0.001, (Figure 1A) indicating that rating differences between the two groups varied with the specific Emotion considered. *Post hoc* tests showed that Intensity ratings from Japanese listeners were significantly lower than ratings from Caucasian listeners for Anger, Disgust, Fear, Surprise, and Pleasure (*t*-test, *p* < 0.05/8: Anger, *t* = −4.358; Disgust, *t* = −4.756; Fear, *t* = −3.073; Surprise, *t* = −2.851; Pleasure, *t* = −6.737: Table 1; Figure 1A).

### Valence

A mixed two-way ANOVA with listeners’ Group (Japanese, Canadian) and Emotion (*n* = 9) as factors was calculated on Valence scores. There was a significant main effect of listeners’ Group: *F*(1, 57) = 5.920, *p* < 0.018, as well as a significant main effect of Emotion *F*(4.3, 244.3) = 224.926, *p* < 0.001 (Greenhouse–Geisser’s test). Crucially, a significant interaction between Group and Emotion was observed: *F*(4.3, 244.3) = 25.101, *p* < 0.001 (Figure 1B) indicating that rating differences between the two groups varied with the specific Emotion considered. *Post hoc* tests showed that Valence ratings from Japanese listeners were significantly higher than ratings from Caucasian listeners for Anger, Disgust, Fear (*t*-test, *p* < 0.05/9: *t*-test, *p* < 0.05/9: Anger, *t* = 6.696, Disgust, *t* = 3.608; Fear, *t* = 3.232: Table 1; Figure 1B), whereas the Valence rating from Japanese listeners was significantly lower than ratings from Caucasian listeners for Pleasure (*t*-test, *p* < 0.05/9; Pleasure, *t* = −8.121; Table 1, Figure 1B).

### Arousal

A mixed two-way ANOVA with listeners’ Group (Japanese, Canadian) and Emotion (*n* = 9) as factors was calculated on Arousal scores. There was no significant main effect of Group: *F*(1, 57) = 2.099, *p* > 0.05, whereas there was a significant main effect of Emotion *F*(4.4, 250.5) = 158.524, *p* < 0.001 (Greenhouse–Geisser’s test). Crucially, a significant interaction between Group and Emotion was observed: *F*(4.4, 250.5) = 8.955, *p* < 0.001 (Figure 1C), indicating that rating differences between the two groups varied with the specific Emotion considered. *Post hoc* tests showed that the Arousal ratings from Japanese listeners were significantly higher than ratings from Caucasian listeners for sad vocalizations (*t*-test, *p* < 0.05/9: sad, *t* = 4.334: Table 1; Figure 1C), whereas the other Emotions were not significantly different between Japanese and Canadian participants (*t*-test, *p* > 0.05/9: Table 1; Figure 1C).

### Sensitivity and specificity

We evaluated the Intensity ratings for their sensitivity (hit rate, by Emotion) and specificity (correct rejection rate, by rating scale). A maximum Intensity rating in the scale corresponding to the portrayed emotion was considered as a hit; otherwise, as a miss. Table 2 shows the Intensity ratings of portrayed emotions for Japanese and Canadian participants: means of hit rates by participants and means of correct rejection rates by participants.

**Table 2:** **Intensity ratings (0–100) averaged across all actors for each portrayed emotion and Intensity ratings scale in Japanese and Canadian participants**.

Intensity rating scale	Portrayed emotion																			Correct rejection rate (%)	
		Neutral		Angry		Disgusted		Fearful		Painful		Sad		Surprised		Happy		Pleased		Specificity	(Validity)
		*M*	SEM	*M*	SEM	*M*	SEM	*M*	SEM	*M*	SEM	*M*	SEM	*M*	SEM	*M*	SEM	*M*	SEM	*M*	SEM
Anger	Japan	9	1.1	**55^bd^**	5.6	18	4.5	25	5.1	33	5.4	14	4.3	21	5.0	7	2.5	12	3.4	36	6.6
	Canada	9	0.5	**75^ac^**	2.4	14	1.1	19	2.1	33	3.8	9	0.7	17	0.8	3	0.3	7	0.8	77	4.4
Disgust	Japan	12	1.3	49	5.7	**45^ac^**	5.9	48	6.3	48	6.2	33	6.2	33	6.0	8	2.9	30	5.8	44*	4.6
	Canada	10	0.5	23	0.7	**70^ac^**	2.7	21	1.6	26	2.7	9	0.6	24	1.4	4	0.3	8	0.7	73*	4.5
Fear	Japan	7	0.8	30	3.7	15	4.0	54	5.9	25	5.5	21	5.1	34	5.9	5	1.7	14	3.2	18*	4.7
	Canada	9	0.5	16	2.0	11	0.6	**68^bc^**	2.5	21	2.0	10	0.7	45	2.6	3	0.2	6	1.0	69*	3.0
Pain	Japan	6	0.8	30	5.7	22	4.7	31	6.1	**52^d^**	5.7	30	6.0	23	5.1	5	1.6	13	3.9	32	8.0
	Canada	9	0.9	24	1.6	11	1.1	31	3.1	**58^ac^**	3.6	26	1.8	21	1.0	3	0.2	7	0.4	62	4.0
Sadness	Japan	10	1.1	15	4.1	23	4.8	21	4.8	27	5.0	**75^ac^**	4.7	13	3.8	7	2.3	26	5.4	75	5.2
	Canada	11	0.8	13	1.2	9	0.8	13	1.2	15	1.5	**77^ac^**	3.6	11	0.4	3	0.2	5	0.3	89	2.5
Surprise	Japan	7	0.8	46	6.2	20	4.5	**66^a^**	5.2	36	6.0	17	4.4	**65^ac^**	5.3	17	4.4	17	4.1	66	7.5
	Canada	9	0.6	26	1.8	26	1.8	57	3.0	35	3.0	11	2.7	**77^ac^**	2.0	18	1.1	25	2.2	64	2.7
Happiness	Japan	7	0.8	12	3.2	13	3.2	9	2.8	9	2.7	13	3.5	15	4.0	**76^ac^**	4.6	25	5.0	59	3.4
	Canada	14	0.5	6	0.4	9	0.8	7	0.4	10	1.1	11	2.4	15	1.3	**81^c^**	1.2	54	3.3	76	3.0
Pleasure	Japan	6	0.8	10	2.9	13	3.3	8	2.4	8	2.4	10	2.7	12	3.2	64	5.6	**32**	6.0	29	5.2
	Canada	13	0.3	6	0.4	9	0.9	6	0.4	11	1.9	10	2.4	12	1.0	76	1.1	**62**	3.8	39	4.0
Hit rate (%)	Japan			44*	7.3	51*	5.1	25*	3.4	35*	7.9	79	4.6	72	6.8	69	3.2	34*	5.7		
	Canada			78*	5.0	81*	3.7	56*	3.0	51*	3.0	86	2.0	75	2.9	60	4.5	59*	3.8		

*Boldface indicates maximum average rating. Note the high hit rates for most affective categories*.

*^a^*p* < 0.001. ^b^*p* < 0.05, strongest rating on the scale corresponding to the portrayed emotion (columns). ^c^*p* < 0.001. ^d^*p* < 0.05, strongest rating for the portrayed emotion corresponding to the rating scale (rows; Fisher’s protected least significance test)*.

*p < 0.05/8, *t*-test.

A Mixed two-way ANOVA with listener’s Group and Emotion (*n* = 8) as factors were calculated on the score of sensitivity and specificity, respectively. In both sensitivity and specificity, a significant main effect of Group was observed [sensitivity: *F*(1, 57) = 51.6, *p* < 0.001; specificity: *F*(1, 57) = 44.8, *p* < 0.001] as well as main effects of Emotion [sensitivity: *F*(5.4, 310) = 38.0, *p* < 0.001; specificity: *F*(5.6, 320) = 41.5, *p* < 0.001, Greenhouse–Geisser’s test]. Interaction effects (Group × Emotion) for sensitivity and specificity were also observed sensitivity: *F*(5.4, 310) = 9.0, *p* < 0.001; specificity: *F*(5.6, 320) = 11.0, *p* < 0.001, indicating that rating differences between the two Groups varied with the specific Emotion considered.

There were significant differences in hit rates between Japanese and Canadian participants for angry, disgusted, fearful, painful, and pleased actors’ vocalizations (*p* < 0.05/8, *t*-test): hit rates for these emotions were all lower in Japanese participants. In correct rejection rate, there were significant differences between Japanese and Canadian participants for Disgust and Fear ratings scales, with lower correct rejection rates in Japanese listeners (*p* < 0.05/8).

In Japanese participants, hit rates for each Emotion varied greatly, from 25% for fearful to 79% for sad. Hit rates and correct rejection rate to happy, sad, and surprised vocalizations were relatively high (more than 50%), whereas hit rates and correct rejection rate to angry, disgusted, fearful, painful, and pleased vocalizations were lower (less than 50%).

In Table 2, the maximum Intensity rating for each portrayed emotion is shown in bold. For fearful vocalizations only, the Emotion with a maximum score by Japanese participants was different from the portrayed emotion. Japanese listeners on average gave higher Intensity rating in the Surprise scale (66%) than the Fear scale (54%) in response to fearful vocalizations. For all other Emotions, Japanese participants gave the maximum ratings in the scale corresponding to the portrayed emotion, as did the Canadian listeners.

### Gender differences of actor and participant

**Figure 2:**
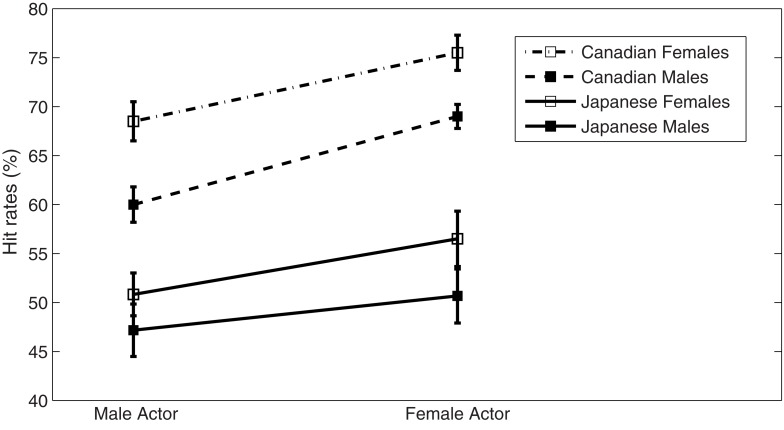
**Hit rates (percentage of test items with maximal rating on the scale corresponding to the portrayed emotion) split by actor’s and participant’s gender**.

We examined the effects of participant’s and actor’s gender on hit rates in Japanese participants (Figure 2). A three-way mixed ANOVA was calculated with the factors of actor’s gender and participant’s gender as well as Emotion in Japanese participants. In addition to a significant effect of the emotion [*F*(1, 56) = 70.285, *p* < 0.001], a significant effect of actor’s gender [*F*(1, 56) = 4.003, *p* ≤ 0.05] was observed, whereas no significant effect was revealed in participant’s gender [*F*(1, 56) = 3.727, *p* > 0.05] or interaction effect: emotion × actor’s gender [*F*(1, 56) < 1, *p* > 0.05], emotion × participant’s gender [*F*(1, 56) = 2.496, *p* > 0.05], and emotion × actor’s gender × participant’s gender [*F*(1, 56) < 1, *p* > 0.05]. Hit rates were higher for vocalizations portrayed by the female actors irrespective of participant’s gender (Figure 2).

Further, we investigated cultural effect on hit rates including Japanese and Canadian participants. A three-way ANOVA was calculated with the factors of listener’s group, actor’s gender, and participant’s gender. A significant main effect was observed in listener’s Group: *F*(1, 110) = 83.211, *p* < 0.001, and actor’s gender *F*(1, 110) = 11.675, *p* < 0.001, and participant’s gender *F*(1, 110) = 8.396, *p* = 0.005 < 0.05. Interaction effect showed no significant effect of listener’s group × participant’s gender, *F*(1, 110) = 0.054, *p* > 0.05, listener’s group × actor’s gender, *F*(1, 110) = 0.428, *p* > 0.05, actor’s gender × participant’s gender *F*(1, 110) = 0.804, *p* > 0.05, and listener’s group × actor’s gender × participant’s gender, *F*(1, 110) = 0.071, *p* > 0.05. These results indicate that in hit rates, the effect of actor’s gender exists regardless of cultures.

Gender differences were analyzed on ratings of Intensity, Valence, Arousal, and correct rejection rates as well as hit rates. A significant effect of actor’s gender was observed in Intensity: *F*(1, 55) = 136.712, *p* < 0.001; Valence: *F*(1, 55) = 14.551, *p* < 0.001; Arousal: *F*(1, 55) = 182.899, *p* < 0.001; correct rejection rates: *F*(1, 55) = 23.131, *p* < 0.001. There was no significant effect of participant’s gender in Intensity: *F*(1, 55) = 0.002, *p* > 0.05; Valence: *F*(1, 55) = 1.289, *p* > 0.05; Arousal: *F*(1, 55) = 0.655, *p* > 0.05. In correct rejection rate, a significant effect of participant’s gender was observed: *F*(1, 55) = 6.343, *p* = 0.015, <0.05. No interaction between actor’s gender and participant’s gender was observed [Intensity: *F*(1, 55) = 1.459, *p* > 0.05, Valence: *F*(1, 55) = 0.316, *p* > 0.05, Arousal: *F*(1, 55) = 2.191, *p* > 0.05, Correct rejection rate: *F*(1, 55) = 0.797, *p* > 0.05].

## Discussion

We investigated cross-cultural differences between Japanese and Canadian participants in their perception of non-verbal affective vocalization using MAVs. The most intriguing finding is that significant Group × Emotion interactions were observed for all emotional ratings (Intensity, Valence, and Arousal). Ratings of Intensity and Valence for happy and sad vocalizations were not significantly different between Japanese and Canadian participants, whereas ratings for angry and pleased vocalizations were significantly different. Especially, for the Valence ratings in angry vocalizations, Japanese subjects rated less negative than Canadian subjects. Further, in the Valence ratings for pleasure vocalizations, Japanese subjects rated less positive than Canadian subjects.

### Cross-cultural effect for positive emotion

Correct rejection rates (validity) of Happiness and Pleasure were not significantly different between Caucasian and Japanese subjects (Table 2: Happiness: Canadian 76% vs. Japanese 56%, Pleasure: Canadian 39% vs. Japanese 29%). These findings suggest that these two items are valid beyond the culture. In our study, there was a significant difference in the ratings (Intensity and Valence) for pleased vocalizations between Japanese and Canadian participants, whereas no significant difference was observed in the ratings for happy vocalizations. Although Happiness (laughter) was well recognized across cultures, there were apparent cultural differences in the perception of Pleasure.

A recent study between Western participants and Namibian participants demonstrated that the positive vocalizations of achievement, amusement, sensual pleasure, and relief were recognized as culture-specific signals although happy vocalizations were recognized cross-culturally (Sauter et al., 2010). Our present result is similar to the findings of this previous study. Further, in accordance with our results, recent studies of facial expression have shown that happy facial expression is not cross-culturally different between Caucasian and Asian participants (Shioiri et al., 1999; Jack et al., 2009, 2012). Our results suggest that the happy emotion is universal in vocal recognition as well as facial recognition. On the other hand, in the vocal recognition, other positive emotions such as Pleasure can show culture-specific biases.

### Cross-cultural effect for negative emotion

Correct rejection rates (validity) of Anger, Pain, Sadness and Surprise were not significantly different between Caucasian and Japanese subjects (Table 2). These findings suggest that these two items are valid beyond the culture. On the other hand, correct rejection rates of Disgust and Fear were significantly different between Caucasian and Japanese subjects (Table 2). These findings indicate that it is very difficult for Japanese to identify these two emotions when they listened to MAV.

A recent cross-cultural study between Western participants and Namibian participants suggested that primary basic negative emotions such as Anger, Disgust, Fear, Sadness, and Surprise can be recognized in both cultures (Sauter et al., 2010). We predicted that ratings of negative emotion are culturally universal. However, our results did not accord with that previous study, and we also observed cross-cultural differences in the recognition of Anger, Disgust, and Fear. Figure 1 and Table 1 show that Intensity ratings for angry, disgusted, fearful, and surprised vocalizations were significantly higher in the Canadian Group than in the Japanese Group. Valence ratings were higher in Japanese than in Canadians regarding some negative emotions (i.e., anger, disgust, and fear). These differences are consistent as higher perceived Intensity of a negative emotion is typically associated with lower (more negative) perceived Valence. These findings could reflect cross-cultural features of Intensity and Valence in negative emotion. Previous studies of facial expression have demonstrated that cross-cultural differences exist in the recognition of angry, disgusted, and fearful face (Shioiri et al., 1999; Jack et al., 2012). In agreement with these results, the recognition of Anger, Disgust, and Fear may reflect cross-cultural differences between Caucasian and Asian participants. On the other hand, the recognition of sad vocalizations (cries) was not significantly different, in agreement with Sauter et al. (2010). Previous studies of facial expression have shown cross-cultural differences in the recognition of sad expressions (Shioiri et al., 1999; Jack et al., 2012). This finding could reflect the fact that the recognition of sad vocalization could be more similar across cultures in comparison with the facial recognition. A previous study indicated that Japanese are severely affected by the meaning of words in recognition of Japanese emotions (Kitayama and Ishii, 2002). The other reason why Japanese find it difficult to differentiate negative emotional vocalizations may be that Japanese need more contextual information to recognize emotions than Canadians.

Concerning of ratings of negative vocalizations, Table 2 shows that hit rates (accuracy) and specificity were lower in Japanese participants than in Canadian participants for ratings of angry, disgusted, fearful, and painful vocalizations. Especially, the strongest pattern of confusion was observed between fearful and surprised vocalizations in Japanese participants. This pattern is a typical pattern of confusion in Caucasian listeners as well (Belin et al., 2008). For both Japanese and Canadian participants, when listening to fearful vocalizations, the Intensity ratings for Surprise were high (Canadian: fearful 68 ± 2.5 vs. surprised 57 ± 3.0; Japanese: fearful 54 ± 5.9 vs. surprised 66 ± 5.2). These results suggest that it was difficult for Japanese participants to discriminate between fearful and surprised vocalizations. The hit rate of fearful vocalizations in Japanese participants was significantly lower than that in Canadian participants. In contrast, the hit rate of surprised vocalizations was not significantly different between Japanese and Canadian. This finding suggests that Japanese tend to be difficult to identify emotional intensity of fearful vocalizations from MAV.

A recent cross-cultural study between Japanese and Dutch participants demonstrated congruency effects displayed by happy face/voice and angry face/voice (Tanaka et al., 2010). This study indicated that, while listening to Anger voices by Dutch speakers, accuracy ratings of Japanese participants are significantly lower than Dutch participants. In agreement with this result, our study showed that ratings for angry vocalizations showed significantly less Intensity and less negative Valence in Japanese than in Canadian listeners.

### The effects of participant’s and actor’s gender in Japanese

Our present study has demonstrated a significant gender effect by actor in accordance with a previous Canadian study (Belin et al., 2008), and hit rates for female vocalizations are higher than for male vocalizations (Figure 2). In general, women are believed to be more emotionally expressive than are men (Fischer, 1993). A previous study of facial recognition also revealed that females had a higher rate of correct classification in comparison with males (Thayer and Johnsen, 2000). Our results suggest that Japanese as well as Canadians are also more accurate at recognizing female vocalizations.

A previous study demonstrated an effect of listener’s gender in Canadian participants (Belin et al., 2008). In line with the previous study, in the analysis including Japanese and Canadian participants, the effect of participant’s gender was replicated.

Our present study has at least two important limitations. First, stimuli consisted of acted vocalizations, not genuine expressions of emotion. Ideally, research on emotional perception would only use naturalistic stimuli. However, collecting genuine emotional expressions across different actors in comparable settings and for different emotions is very difficult and presents ethical problems. Second, in the present study, cross-cultural differences between Canadian and Japanese listeners were confirmed in the recognition of some emotional vocalizations. In the future, it will be necessary to develop a set of stimuli to increase cross-cultural validity.

In summary, we tested for cross-cultural differences between Japanese and Canadian listeners in perception of non-verbal affective vocalization using MAVs. Significant Group × Emotion interactions were observed for all ratings of Intensity, Valence, and Arousal in comparison with Japanese and Canadian participants of our present study. Although ratings did not differ across cultural groups for Pain, Surprise, and Happiness, they markedly differed for the angry, disgusted, and fearful vocalizations which were rated by Japanese listeners as significantly less intense and less negative than by Canadian listeners; similarly, pleased vocalizations were rated as less intense and less positive by Japanese listeners. These results suggest, in line with Sauter et al. (2010), that there were cross-cultural differences in the perception of emotions through non-verbal vocalizations, and our findings further suggest that these differences are not necessarily only observed for positive emotions.

## Conflict of Interest Statement

The authors declare that the research was conducted in the absence of any commercial or financial relationships that could be construed as a potential conflict of interest.
